# The Effect of Chronic Administration of Saffron (*Crocus sativus*) Stigma Aqueous Extract on Systolic Blood Pressure in Rats

**DOI:** 10.17795/jjnpp-12475

**Published:** 2013-11-01

**Authors:** Mohsen Imenshahidi, Bibi Marjan Razavi, Ayyoob Faal, Ali Gholampoor, Seyed Mehran Mousavi, Hossein Hosseinzadeh

**Affiliations:** 1 Department of Pharmacodynamy and Toxicology, Pharmaceutical Research Center, School of Pharmacy, Mashhad University of Medical Sciences, Mashhad, IR Iran; 2 School of Pharmacy, Mashhad University of Medical Sciences, Mashhad, IR Iran

**Keywords:** Crocus, Blood Pressure, Desoxycorticosterone

## Abstract

**Background:**

*Crocus sativus* L. (saffron), which belongs to the Iridaceae family, is widely cultivated in Iran. Cardiovascular effects of saffron has been established in some studies but the effects of chronic administration of saffron (*C. sativus*) stigma aqueous extract on blood pressure has not been investigated.

**Objectives:**

In this study the effects of saffron (*C. sativus*) stigma aqueous extract on blood pressure of normotensive and desoxycorticosterone acetate (DOCA)-salt induced hypertensive rats, in chronic exposure was evaluated.

**Materials and Methods:**

Five weeks administration of three doses saffron aqueous extract (10, 20 and 40 mg/Kg/day) and spironolactone (50 mg/Kg/day) in different groups of normotensive and hypertensive rats (at the end of 4 weeks treatment by DOCA-salt) was carried out and their effects on mean systolic blood pressure (MSBP) and heart rate (HR) were evaluated using tail cuff method. The duration of the effect of saffron on systolic blood pressure (SBP), was also evaluated.

**Results:**

Our results indicated that chronic administration of saffron aqueous extract could reduce the MSBP in DOCA salt treated rats in a dose dependent manner. This compound did not decrease the MSBP in normotensive rats. The data also showed that antihypertensive effects of saffron did not persist.

**Conclusions:**

It is concluded that saffron aqueous extract possesses antihypertensive and normalizing effect on BP in chronic administration.

## 1. Background

*Crocus sativus* L. (saffron) is a perennial stem less herb which belongs to the Iridaceae family. It is widely cultivated in Iran and other countries. Major components including volatile agents (e.g. safranal), bitter principles (e.g. picrocrocin) and dye materials (e.g. crocetin and its glycoside, crocin) are considered as the pharmacologically active components of saffron (1). In traditional medicine, as well as in modern pharmacology, saffron has been used in the treatment of numerous diseases. It was reported that *C. sativus* L. and its constituents have antitumor (2), anti-inﬂammatory, antinociceptive (3), antioxidant (4), antidepressant (5), hypolipidemic (6) and anticonvulsant effects (7), and could improve memory as well learning abilities in rats (8, 9). Saffron and its active components also showed protective effects on diazinon and acrylamide induced oxidative stress (10-12). Evidence showed that saffron and its constituents reduced lipid peroxidation in various tissues including kidney (13), hippocampal (14), muscle skeletal (15) and heart (16) following oxidative damages in rats. Furthermore radical scavenging effect of *C. sativus* L. extract and its bioactive constituents, safranal and crocin have been shown previously using DPPH (1,1-diphenyl-2-picryl-hydrazyl) radical scavenging test (17), deoxyribose assay and microsomal lipid peroxidation induced by Fe^2+^/ascorbat (4).

Cardiovascular effects of saffron and its components have been established in some studies (16). It was reported that saffron aqueous extract may have cardioprotective effects in isoproterenol induced myocardial infarction through modulation of oxidative stress in such a way that it maintains the redox status of the cell (16). Moreover it was established that aqueous-ethanol extract of *C. sativus*, possesses a potent inhibitory effect on heart rate and contractility of guinea pig heart via calcium channel-blocking effect (18). Also in another study the hypotensive effect of *C. sativus* petals extract in rats has been shown (19). The results of our previous study showed that the aqueous extract of saffron stigma as well as two major constitutes of this plant, namely crocin and safranal, has hypotensive properties in normotensive and hypertensive anaesthetized rats (20).

## 2. Objectives 

Although the effect of this plant in lowering blood pressure have been shown previously, but there has not been any study about the effect of saffron on blood pressure through chronic administration. Thus, in this study the effects of chronic administration of saffron stigma aqueous extract, on blood pressure of normotensive and desoxycorticosterone acetate (DOCA)-salt induced hypertensive rats were investigated.

## 3. Material and Methods

### 3.1. Animal and Chemicals

Adult male Wistar rats (weight 250–300 g) were provided by animal center (School of Pharmacy, Mashhad University of Medical Sciences). They were maintained on a 12 hours light/dark cycle and at a temperature of 23 ± 1˚C with free access to food and water. These conditions were maintained constant throughout the experiments. The experiments were performed under the Animals (scientiﬁc procedures) Act of 1986 and conform to the National Institutes of Health guidelines for the use of experimental animals. The aqueous extract was dissolved in saline (0.9% NaC1). Saline (0.9% NaCl) was used as negative control. DOCA was purchased from Iran Hormone.

### 3.2. Plant and Extracts 

*C. sativus* L. stigma were collected from Ghaen (Khorasan province, northeast Iran) and analyzed in accordance to the ISO/TS 3632-2. Aqueous extract of *C. sativus* was prepared by maceration method. Briefly, 8 g of stigma powder was macerated in 300 mL distilled water for 72 hours with continuous shaking in the refrigerator. Supernatant was separated by centrifuging and transferred to a freeze-drier. After 24 hours, lyophilized powder of extract was available.

### 3.3. Induction of Experimental Hypertension

Desoxycorticosterone acetate (DOCA)-salt (20 mg/Kg, twice weekly, for 4 weeks, s.c.) and NaCl (1%) in rat’s drinking water were used for induction of hypertension ( 20 ). Rats were randomly divided into 7 groups: 1) Saline injected (0.5 mL/Kg, twice weekly, s.c., for 4 weeks), this treatment was continued for another five weeks; 2) (DOCA)-salt (20 mg/Kg, twice weekly, for 4 weeks, s.c.), DOCA treatment was continued by intraperitoneal injection (i.p. injection) of 0.5 mL/Kg normal saline for another five weeks; 3, 4 and 5) (DOCA)-salt (20 mg/Kg, twice weekly, for 4 weeks, s.c.), DOCA treatment was continued by i.p. injection of 10, 20 and 40 mg/Kg/day saffron stigma aqueous extract for another five weeks, after that saffron aqueous extract injection was stopped but DOCA injection was continued for another two weeks; 6) (DOCA)-salt (20 mg/Kg, twice weekly, for 4 weeks, s.c.), DOCA treatment was continued by i.p. injection of 50 mg/Kg/day spironolactone for another five weeks, after that spironolactone injection was stopped but DOCA injection was continued for another two weeks; 7) Saline injected (0.5 mL/Kg, twice weekly, s.c., for 4 weeks), saline treatment was continued by i.p. injection of 40 mg/Kg/day saffron stigma aqueous extract for another five weeks. All groups consisted of six rats. Table 1 describes the different groups that were selected for this study.

**Table 1.** Summary of Selected Groups

Groups	DOCA (9 Weeks)	Normal Saline (9 Weeks)	Normal saline (4^th^ to 9^th^ Weeks)	Aqueous saffron extract (4^th^ to 9^th^ Weeks)	Spironolactone (4^th^ to 9^th^ Weeks)
**1**		*			
**2**	*		*		
**3, 4, 5 **	*			*	
**6**	*				*
**7**		*		*	

### 3.4. Hypotensive Activity

Four, nine and eleven weeks after the ﬁrst saline or DOCA treatment, SBP was measured using tail cuff method in all groups as described by Lorenz (21). Briefly, three days before the last treatment, the training of rats in different groups for indirect SBP measurements was started. This training consisted of the regular handling of the animals and getting used to the restraining cage and the tail-cuff. Rats were heated for approximately 15 minutes at 30-32˚C to increase blood flow to the tail. After that, animals were placed in small restraining cages with a cuff around the end of proximal of the tail. After placing of the cuff, a pulse transducer was used around the end of the tail. Then the tail cuff was inflated using the related button on the Non-invasive blood pressure (NIBP) controller apparatus and Acquisition data were performed by a computerized system power lab (ADInstruments, v 5.4.2). The mean values of the five blood pressure (BP) and heart rate (HR) readings were used for each animal.

### 3.5. Statistical Analysis

Data are expressed as Mean ± SEM. Statistical analysis was performed using one-way ANOVA followed by the Tukey-Kramer post-hoc test for multiple comparisons. P-values less than 0.05 were considered statistically significant.

## 4. Results

### 4.1. Effect of DOCA on SBP

**Figure 1.**
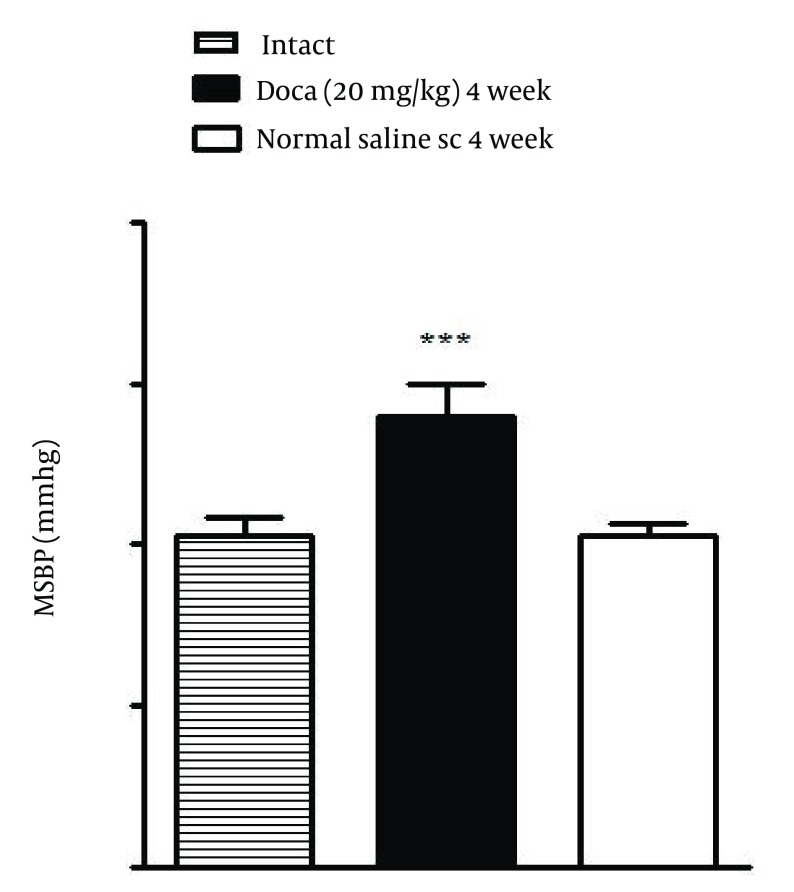
Hypertension Induced by Desoxycorticosterone Acetate (DOCA)-salt After 4 Weeks Each value is the Mean ± SEM of six experiments, *** P < 0.001 vs normal saline treated rats. One way ANOVA, Tukey Krumer test.

In DOCA treated rats, MSBP significantly increased in comparison with normal saline treated (normotensive) rats (P < 0.001) (Figure 1).

### 4.2. Effects of Aqueous Extract in Normotensive and Hypertensive Rats After Nine Weeks 

**Figure 2.**
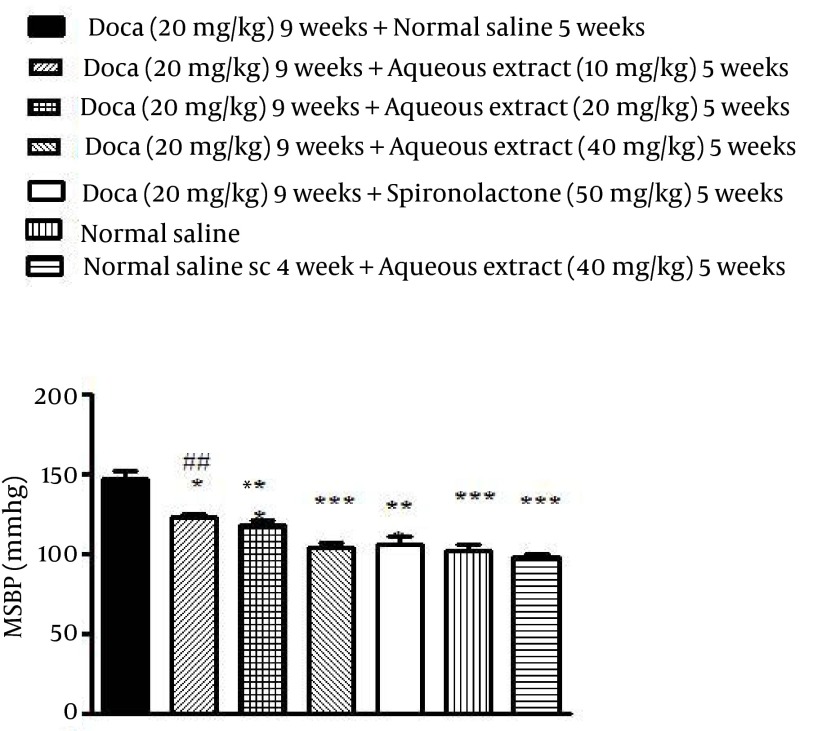
Mean Systolic Blood Pressure (MSBP) in Response to Various Doses of *Crocus sativus* stigma Extract in Normotensive and Hypertensive Rats at the End of Nine Weeks Each value is the Mean ± SEM of six experiments. One-way ANOVA, Tukey Krumer, * P < 0.05, ** P < 0.01, *** P < 0.001 vs DOCA plus normal saline treated rats, ## P < 0.01 vs DOCA plus spironolactone treated rats.

As shown in Figure 2 the injection of aqueous extract (10, 20 and 40 mg/Kg) reduced the MSBP in hypertensive animals (P < 0.05, P < 0.0 1 and P < 0.001, respectively), dose dependently. In normotensive rats, aqueous extract did not reduce the MSBP. The hypotensive effect of aqueous extract in the highest dose was similar to that of spironolactone.

### 4.3. The Evaluation of Duration Effect of Saffron Aqueous Extract on SBP

**Figure 3.**
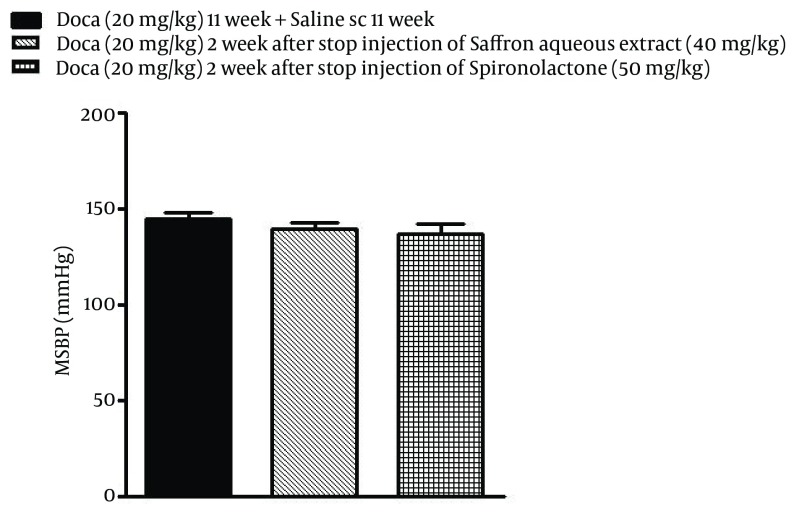
Evaluation of the Duration Hypertensive Effect of Saffron Aqueous Extract Each value is the Mean ± SEM of six experiments. One-way ANOVA, Tukey Krumer.

As shown in Figure 3, the decreasing level of SBP at the highest doses of saffron aqueous extract as well as spironolactone did not persist in rats and after stopping the administration, SBP increased again at the end of eleven weeks.

## 5. Discussion

In this study we attempted to evaluate the effects of chronic exposure to saffron (*C. sativus*) stigma aqueous extract on the blood pressure of normotensive and desoxycorticosterone acetate (DOCA)-salt induced hypertensive rats. Deoxycorticosterone acetate (DOCA)-salt is an agent commonly used to induce hypertension in experimental animals (20). Our results showed that DOCA-salt significantly induced hypertension in comparison with saline group at the end of 4 weeks treatment. Chronic administration of aqueous extract of saffron (*C. sativus*) stigma reduced the increase of MSBP induced by DOCA, but this hypotensive effect was not observed in normotensive rats. Previous studies revealed that saffron and its constituents possess vasodilatory effects. For example, a potent relaxant effect of *C. sativus* and safranal on smooth muscles of guinea pigs has been shown (22). Hence, it might be concluded that hypotensive effect of saffron in chronic treatment is related to the inhibitory effect on smooth muscles via blocking calcium channel or inhibiting sarcoplasmic reticulum Ca^2+^ release into the cytosol. Also, it was shown that aqueous and ethanolic extracts of saffron petals, reduced the mean arterial blood pressure (MABP) in anaesthetized rats (22). Moreover it was indicated that intravenous injection of aqueous extract of saffron stigma (2.5, 5 and 10 mg/Kg) and two major constitutes of this plant have hypotensive effects in normotensive as well hypertensive anaesthetized rats in a dose-dependent manner (23). In this study, the reflex tachycardia was not observed (data not shown), so it could be suggested that both heart function and blood vessels contractility are affected by saffron (23). Based on pathophysiological and biochemical changes followed by administration of DOCA-salt in rats, it is believed that DOCA-salt hypertensive rats, provides an animal model of oxidative and inflammatory stress in the cardiovascular system (23). So, the DOCA-salt experiment can provide an appropriate model to evaluate anti-oxidative or anti-inflammatory responses of natural or synthetic compounds on cardiovascular system. This also provides opportunities for the development of novel therapeutic agents for the management of chronic cardiovascular disease (23). The preventive effects of some antioxidants on hypertension and oxidative stress induced by deoxycorticosterone acetate (DOCA)-salt have been established previously. For example, quercetin showed both antihypertensive and antioxidant properties in the model of (DOCA)-salt induced hypertension in chronic treatment (24). As saffron is an essential source of antioxidants such as crocin, it could be concluded that the antihypertensive effects of saffron could be related partly to its antioxidant properties (4). It is well known that DOCA induced hypertension causes an endothelial dysfunction in the isolated aortic rings as well as in the perfused mesenteric bed (25). As saffron aqueous extract decreased SBP in hypertensive rats, our results may also show that the vasodilatory effects of saffron were endothelium dependent. Spironolactone, known as potassium-sparing diuretics, inhibits the effects of aldosterone by competing for intracellular mineralocorticoid receptors in the cortical collecting duct. This decreases the reabsorption of sodium and water, as well the secretion of potassium (26). In this study spironolactone was used as a positive control. Our results showed that the antihypertensive effect of aqueous extract of saffron at the highest dose was as much as spironolactone at the end of nine weeks. It is likely that the hypotensive effect of saffron may be due to the diuretic effect of this plant (1). To evaluate the duration of effects of saffron on reducing SBP, the injection of saffron was stopped at the end of nine weeks but DOCA injections were continued for another two weeks. The data showed that antihypertensive effects of saffron did not persist, so it could be postulated that long term blood pressure regulation systems were not affected by saffron.

In summary our results indicated that chronic administration of saffron aqueous extract could reduce the MSBP in DOCA salt treated rats. So saffron possesses antihypertensive and normalizing effect on BP.
